# Impact of High-Dose Multi-Strain Probiotic Supplementation on Neurocognitive Performance and Central Nervous System Immune Activation of HIV-1 Infected Individuals

**DOI:** 10.3390/nu9111269

**Published:** 2017-11-21

**Authors:** Giancarlo Ceccarelli, Jason M. Brenchley, Eugenio Nelson Cavallari, Giuseppe Corano Scheri, Mariangela Fratino, Claudia Pinacchio, Ivan Schietroma, Saeid Najafi Fard, Carolina Scagnolari, Ivano Mezzaroma, Vincenzo Vullo, Gabriella d’Ettorre

**Affiliations:** 1 Department of Public Health and Infectious Diseases, University of Rome “Sapienza”, Rome (Italy) and Azienda Policlinico Umberto I, 00161 Rome, Italy; eugenionelson.cavallari@uniroma1.it (E.N.C.); giuseppe.coranoscheri@uniroma1.it (G.C.S.); claudiapinacchio@gmail.com (C.P.); schietroma.ivan@gmail.com (I.S.); saeid.najafifard@gmail.com (S.N.F.); vincenzo.vullo@uniroma1.it (V.V.); gabriella.dettorre@uniroma1.it (G.d.E.); 2 Laboratory of Parasitic Diseases, National Institute of Allergy and Infectious Diseases, NIH, Bethesda, MD 20892, USA; jbrenchl@niaid.nih.gov; 3 Department of Neurology, University of Rome “Sapienza”, 00185 Rome, Italy; marifra@hotmail.it; 4 Department of Molecular Medicine, Laboratory of Virology, University of Rome “Sapienza”, 00185 Rome, Italy; carolina.scagnolari@uniroma1.it; 5 Department of Clinical Medicine, University of Rome “Sapienza”, 00185 Rome, Italy; ivano.mezzaroma@uniroma1.it

**Keywords:** HIV, asymptomatic neurocognitive impairment, immune activation, central nervous system, neopterin, probiotic, supplementation, multi-strain, dysbiosis, gut-brain axis

## Abstract

**Background:** Gut microbiota has metabolic activity which influences mucosal homeostasis, local and systemic immune responses, and other anatomical systems (i.e., brain). The effects of dysbiosis are still poorly studied in Human Immunodeficiency Virus-1 (HIV-1) positive subjects and insufficient data are available on the impairment of the gut-brain axis, despite neurocognitive disorders being commonly diagnosed in these patients. This study evaluated the impact of a probiotic supplementation strategy on intrathecal immune activation and cognitive performance in combined antiretroviral therapy (cART) treated HIV-1 infected subjects. **Methods:** Thirty-five HIV-1 infected individuals were included in this study. At baseline (T0) a battery of tests was administered, to evaluate neurocognitive function and a lumbar puncture was performed to determine neopterin concentration in cerebrospinal fluid (CSF), as a marker of Central Nervous System (CNS) immune activation. Subsequently, a subgroup of participants underwent a 6-month course of multi-strain probiotics supplementation; this intervention group was evaluated, after probiotic treatment, with a second lumbar puncture and with repeated neurocognitive tests. **Results:** At T0, all participants showed impaired results in at least one neurocognitive test and elevated neopterin concentrations in CSF. After supplementation with probiotics (T6), the interventional group presented a significant decrease in neopterin concentration and a significant improvement in several neurocognitive tests. In contrast, no significant modifications were observed in the neurocognitive performance of controls between T0 and T6. The CNS Penetration Effectiveness Score of antiretroviral therapy did not show an influence from any of the investigated variables. **Conclusions:** Multi-strain probiotic supplementation seems to exert a positive effect on neuroinflammation and neurocognitive impairment in HIV-1 infected subjects, but large trials are needed to support the concept that modulation of the gut microbiota can provide specific neurological benefits in these patients.

## 1. Introduction

Following the widespread use of combined antiretroviral therapy (cART) the prevalence of severe neurocognitive impairments, among HIV-1 infected individuals, considerably decreased, while the prevalence of milder extents of HIV-1 associated neurocognitive disorders, such as Asymptomatic Neurocognitive Impairment, persisted at a stable rate among this population [1]. Wider evidence shows that changes in the qualitative and quantitative composition of the gut’s microbiome can affect the modulation of the gut-brain axis, thus resulting in the onset of behavioral and neurocognitive alterations [2]. For these reasons, the use of probiotics represents a novel potential approach to manage conditions, such as stress-related behaviors, and to ameliorate cognitive function in several pathological settings [3,4].

HIV-1 infected patients usually suffer from two conditions that negatively affect neurocognitive function: the alteration of the normal gut flora composition (dysbiosis), with the overgrowth of detrimental bacterial strains [5] and elevated cerebrospinal fluid (CSF) concentrations of neopterin, a biomarker of Central Nervous System (CNS) immune activation [6]. Neopterin—a biochemical product of the guanosine triphosphate pathway—is a recognized marker of monocyte activation, and an association between its expression and the development of HIV-associated neurocognitive disorders (HAND), Acquired Immuno-Deficiency Sindrome (AIDS) dementia complex, and HIV encephalitis have been proposed [7,8,9,10]. The expression of neopterin has been found to be higher in HIV-1-positive patients, compared to healthy people, and in naïve HIV-1-positive patients, compared to those on effective cART [10,11]. In this study, we evaluated the impact of a 6 month course of high dose multi-strain probiotic supplementation on CSF immune activation and neurocognitive impairment of HIV-1 positive patients.

## 2. Materials and Methods

### 2.1. Study Design, Recruitment, Study Eligibility Criteria and Ethics Statement

The present study included 35 HIV infected individuals, enrolled at the HIV Outpatient Clinic of the Department of Public Health and Infectious Diseases of University of Rome “Sapienza”. The study protocol was approved by the internal committee of the Department of Public Health and Infectious Diseases of “Sapienza” University of Rome and by the Ethics Committee of Policlinico Umberto I Hospital, Rome (ethical approval code Rif# 2970). All participants agreed to the enrolment by signing a written informed consent form.

**Figure 1:**
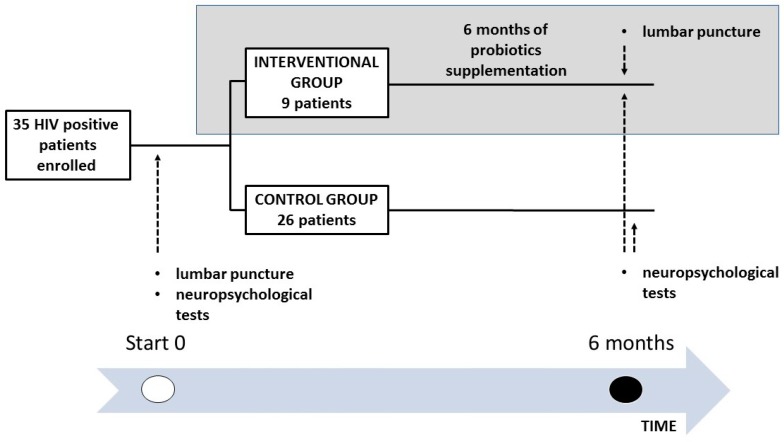
The design of the study. (Abbreviations. HIV: Human Immunodeficiency Virus).

The design of the study is shown in Figure 1. Inclusion criteria for the enrolment in the study were: age >18 years old, a stable and effective (plasma HIV RNA <37 copies/mL) cART regimen for at least 6 months prior to enrolment. Exclusion criteria were: education below primary school, Mini Mental State Examination <26, any impairment in daily living activities, as defined in the Instrumental Activities of Daily Living scale, previous history or actual diagnosis of any neurologic or psychiatric condition, positive Polymerase Chain Reaction (PCR) on CSF for any of the following pathogens: Cytomegalovirus (CMV), Epstein-Barr virus (EBV), Herpes simplex virus-1 (HSV-1), Herpes simplex virus-2 (HSV-2), Varicella-zoster virus (VZV), Human herpesvirus-8 (HHV-8), BK virus (BKV), JC virus (JCV). At baseline (T0) all participants underwent (I) a lumbar puncture, to assess HIV-RNA, PCR for CMV, EBV, HSV-1, HSV-2, VZV, HHV-8, BKV, JCV, and neopterin concentration in CSF, (II) an array of neuropsychological tests.

Subsequently, we divided the enrolled patients into two groups, on the basis of their neuroinflammation levels (interventional group with higher level of CSF neopterin and control group with lower levels of CSF neopterin). Nine participants with higher levels of CSF neopterin (interventional group) underwent a six month course of supplementation with oral probiotics (2 sachets, each containing 450 × 10^9^ billion bacteria, twice a day); the selected probiotic was a commercially available product with the following composition: *Lactobacillus plantarum* DSM 24730, *Streptococcus thermophilus* DSM 24731, *Bifidobacterium breve* DSM 24732, *Lactobacillus paracasei* DSM 24733, *Lactobacillus delbrueckii* subsp. *bulgaricus* DSM 24734, *Lactobacillus acidophilus* DSM 24735, *Bifidobacterium longum* DSM 24736, and *Bifidobacterium infantis* DSM 24737 (Vivomixx^®^, Dupont, Madison, WI, USA).

At the end of the supplementation period (T6) participants from the intervention group underwent a second lumbar puncture and a second neurocognitive assessment battery, while controls (the remaining 26 subjects) were assessed with a second assay of neurocognitive tests (a parallel version of the Rey Auditory Verbal Learning Test (RAVLT) was administered at T6; parallel versions with Italian validation for the other tests are not available).

### 2.2. Neuropsychological Test Battery

Neuropsychological tests, administered by a trained neuropsychologist, explored verbal areas, language, attention, working memory, abstraction, executive, learning memory, processing speed of information, sensory-perceptual and motor skills. The tests included the Rey–Osterrieth Complex Figure Test (ROCF), to evaluate participants’ recognition and recall skills for non-verbal contents, the Rey Auditory Verbal Learning Test (RAVLT) to evaluate short term auditory-verbal memory, rate of learning and retention of information, the Test of Weights and Measures Estimation (STEP), to evaluate abstraction skills, the Visual Search Test (Attention Matrices Test) to evaluate attention skills, the Verbal Fluency test (FAB), to evaluate executive functions and the ability to switch between different tasks, the Test of Phonological and Semantic Verbal Fluency (respectively PVF and SVF) to evaluate phonological and semantic supplies and the ability to access them, Raven’s Standard Progressive Matrices (SPM), to evaluate abstract reasoning and problem solving capabilities, the Digit Span test, to evaluate short term memory and executive functions, the Corsi Block Tapping Test (CBTT) to evaluate short term spatial memory and executive functions, the Aachener Aphasia Test (AAT), to evaluate the presence of aphasia among study participants, the Trail Making Test A and B (TMT A and TMT B), to evaluate visual-spatial attention and motor skills. On average, the neuropsychological evaluation required 45 min for the operator.

### 2.3. Evaluation of Neopterin Levels by ELISA Assay

CSF was collected by lumbar puncture, and cell-free centrifuged supernatant samples were stored at −80 °C. CSF neopterin levels were determined by a commercially available solid phase enzyme-linked immunosorbent assay (ELISA), based on the basic principle of a competitive ELISA (IBL International GmbH, Hamburg, Germany). The upper normal reference value was previously determined to be 5 nmol/L (upper limit of the 99% confidence interval) [12].

### 2.4. Bacterial DNA Isolation from Fecal Samples

Faecal samples from patients enrolled in the intervention group, were collected at T0 and T6, in order to evaluate adherence to the treatment and the efficacy of the multi-strain probiotic supplementation in changing the microbiota composition. For this reason, the QIAamp DNA Stool Mini Kit (Qiagen, Hilden, Germany) was used according to the manufacturer’s instructions: 200 mg of frozen samples were suspended in 1.4 mL of ASL lysis buffer from the stool kit, added with glass beads (150–212 μm, Sigma–Aldrich, St. Louis, MO, USA), and homogenized. The suspension was incubated at 95 °C for 5 min, DNA was purified and eluted in 200 μL of AE buffer and the samples obtained were stored at −20 °C. Finally, bacterial DNA from faecal samples was extracted and quantified by a real-time PCR, performed to evaluate Bifidobacteria levels. Briefly, PCR amplification and detection were performed on optical-grade 96-well plates, using the Applied Biosystems 7500 Real-Time PCR instrument (Applied Biosystems, Inc., Norwalk, CT, USA). The reaction mixture (25 μL) was composed of SensiMix SYBR Low-ROX (BIOLINE, Taunton, MA, USA), 500 nM primers for *Bifidobacterium* genus, and 2.5 μL of template DNA. A melting curve analysis was made after amplification, to distinguish target amplicons from aspecific non-target PCR products. Standard curves were made by using 10-fold dilutions of DNA, extracted from *Bifidobacterium breve*. All samples were analyzed in duplicate in two independent real-time PCR assays.

### 2.5. Statistical Analysis

A Wilcoxon test for paired samples and Pearson’s correlation coefficient were applied for data analysis, using SPSS version 24 for Windows (IBM, New York, NY, USA). Graphics were done using GraphPad Prism software, version 5.0 (GraphPad Software Inc., La Jolla, CA, USA). Differences were considered statistically significant when *p* < 0.05.

## 3. Results

### 3.1. Demographic and Clinical Characteristics of HIV-1-Positive Patients

All 35 HIV-1 infected individuals enrolled were Caucasian. The majority of our population was represented by males (94%), the median age of participants was 48 years old (IQR: 38–54) and the median duration of time from diagnosis was 14 years (IQR: 7–23). All included subjects had finished at least the primary course of school education.

Individuals enrolled in the study had been taking cART therapy for a median of 14 years (IQR: 8–19) and they had been on a stable and effective ARV regimen for at least 1 year at the time of inclusion (all participants showed plasma HIV RNA <37 copies/mL at enrollment). Antiretroviral therapy did not change and anti-inflammatory drugs were not used during the follow-up. The median value, in regard to cluster of differentiation 4 (CD4) nadir, was 250 cell/μL (IQR: 45–400) while the median value of the actual CD4 count was 566 cell/μL (IQR: 397–714).

The CNS Penetration-Effectiveness score (CPE) of ARV regimens included in the study was calculated according to the classification proposed by Letendere et al. and the median CPE score of our population was 7 (IQR: 7–8).

### 3.2. Correlations between Neuroinflammation and Neuropsychological Impairment

At baseline (T0), all participants underwent a lumbar puncture to evaluate their HIV-1 viral load and the extent of neuro-inflammation (determined through the concentration of neopterin) into CSF. All subjects enrolled in the study showed an HIV-RNA in CSF <37 copies/mL and the median concentration of neopterin in CSF was 23.4 nmol/L (10.5–65.2). Participants also showed negative polimerase-chain reaction (PCR) tests on CSF for CMV, EBV, HSV-1, HSV-2, VZV, HHV-8, BKV and JCV.

At baseline, all participants showed a normal performance on the Mini-Mental State Examination (MMSE) and no impairment on the instrumental activities of daily living (IADL) scale. Neuropsychological tests, administered at T0, with the aim of assessing the neurocognitive performance, showed that all subjects presented an altered result in at least one test exploring the executive functions, moreover most participants presented with a pathological impairment in at least two different domains.

In our population, we did not observe a correlations between the CPE score and neopterin concentration in CSF (*r* = 0.220; *p* = 0.271); the CPE score did not show any correlations with the results of any of the proposed neurocognitive tests either. On the contrary, at T0, neopterin was inversely correlated with the results for the following tests: forward Corsi Block Tapping Test (*r* = −0.474; *p* = 0.004), backward Corsi Block Tapping Test (*r* = −0.468; *p* = 0.005), forward Digit test (*r* −0.480; *p* = 0.004) and Verbal Fluency test (*r* = −0.361; *p* = 0.033). Neopterin showed slight inverse correlations with Raven’s Standard Progressive Matrices test (*r* = −0.308; *p* = 0.071), the time estimation during the Test of Weights and Measures Estimation (*r* = −0.295; *p* = 0.085) and the Test of Weights and Measures total score (*r* = −0.294; *p* = 0.087).

### 3.3. Results of Probiotic Supplementation: Reduction of Neuroinflammation and Recovery of Neuropsychological Impairment

A subgroup of nine subjects (intervention group), including the individuals presenting with the highest extent of neuro-inflammation from the 35 patients enrolled, underwent a 6 months course with a high dose of oral probiotics supplementation (the main characteristics of this subpopulation are shown in Table 1). The main characteristics of this subpopulation of nine people, compared with the control group of the remaining 26 subjects, are shown in Table 1.

The median neopterin concentration in CSF at T0 in this subpopulation was 34.14 nmol/L (IQR: 22.53–65.2), showing no significant difference in comparison to the median value of the entire population (*p* = 0.655), but significantly higher than the median value of the control group (*p* = 0.008) Before probiotic supplementation (T0), we did not find significant differences in neurocognitive performances between the two subgroups, despite the differences in neopterin levels. (Table 2). No statistically significant difference in CSF neopterin levels were found between the two groups (*p* > 0.05) at T0; higher levels were found in patients with the impairment of at least two neurocognitive domains, than in those with a single neurocognitive domain impairment.

At the end of supplementation (T6), in the intervention group, we observed that the results of viral replication in CSF remained stably suppressed (HIV-RNA in CSF resulted <37 copies/mL in all subjects at T6) and the neopterin concentration in CSF significantly decreased at T6 when compared to T0 values (T6 24.11 nmol/L vs. T0 34.14 nmol/L; *p* = 0.011). The results of the neuropsychological tests at T0 and T6 among participants who underwent probiotics supplementation are shown in Table 3; an improvement in overall neurocognitive performance was observed in the majority of our population, with a significant improvement revealed in multiple tests—immediate copy of the Rey–Osterrieth Complex Figure (*p* = 0.0089), delayed copy of the Rey–Osterrieth Complex Figure (*p* = 0.0039), immediate recall during the Rey Auditory Verbal Learning Test (*p* = 0.027), delayed recall during the Rey Auditory Verbal Learning Test (*p* = 0.042), time estimation during the Test of Time and Weights Estimation (*p* = 0.043), weight estimation during the Test of Time and Weights Estimation (*p* = 0.035), Phonological Verbal Fluency Test (*p* = 0.027), Trail Making Test A (*p* = 0.0502) and forward Corsi Block Tapping Test (*p* = 0.057). A direct correlation with neopterin concentration in CSF was observed at T6 with the results for the Trail Making Test B (*r* = 0.741; *p* = 0.022); in contrast an inverse correlation was observed between neopterin and the forward Corsi Block Tapping Test (*r* = −0.793; *p* = 0.011). A slight inverse correlation was also observed between Raven’s Standard Progressive Matrices test and neopterin in CSF (*r* = −0.644; *p* = 0.061). CPE scores did not show any correlation with neopterin or any of the provided neurocognitive tests, at T6. No difference was observed in the CD4 count between T0 and T6 among these individuals (674 cell/μL vs. 682 cell/μL; *p* = 0.959).

Neurocognitive performance was also evaluated among the 26 individuals who did not undergo supplementation with probiotics; this was assessed by a second administration of the assay of neuropsychological tests at T6 (results are shown in Table 3). No difference between T0 and T6 was observed for neurocognitive performance among this group of participants.

### 3.4. Adherence to Probiotic Supplementation and Safety of the Treatment

Adherence to the probiotic supplementation was documented by the increase in Bifidobacteria spp. in fecal samples collected at T6, compared to their basal level (T0). No side effects were observed over the course of 6 months of treatment in all patients.

## 4. Discussion

In recent years, the interdependence between the microbiome, gut and brain has been highlighted. The disruption of the gut mucosa barrier and intestinal dysbiosis—both conditions commonly observed in HIV-1 infected patients—play a pivotal role in the pathogenesis of HIV infection [13] and several studies have shown the beneficial effects of probiotics on the intestinal barrier, systemic immune activation and tryptophan metabolism of on HIV-1 infected patients [14,15].

At T0, in the overall analysis of the 35 patients enrolled, we observed that higher neopterin levels in CSF were correlated with a poorer result in the neurocognitive tests. No correlation was observed between the CNS Penetration Effectiveness (CPE) score and the neopterin concentration in CSF, nor between the CPE score and neurocognitive performance, suggesting a possible low impact of cART on the treatment of neuroinflammation and neurocognitive impairment.

When we divided the enrolled patients into two groups (intervention group and control group) on the basis of their neuroinflammation levels, we observed that, at T0, both groups showed similar results in neurocognitive performances (Table 2), despite their different neopterin concentrations observed in CSF; taken together, these data suggest that the levels of neuroinflammation are not the only determinant of the neurocognitive impairment in HIV-1 infected patients. On the other hand, to investigate the role of dysbiosis on neuroinflammation and cognitive performance, we assigned nine participants (with highest levels of neuroinflammation shown by their levels of neopterin in CSF, which were higher than the control group) from our study population, to start supplementation with oral probiotics for 6 months (interventional group).

At the end of the supplementation period, we observed a reduction in CNS immune activation and an improvement in cognitive performance in the intervention group, thus suggesting a possible role of probiotics in the treatment of the intrathecal immune activation and cognitive impairment of HIV infected individuals. In fact, at T6, the intervention group showed improved results in comparison to controls in the following tests: immediate recall of ROCF, delayed recall of ROCF, immediate recall of RAVLT, delayed recall of RAVLT, recognition of RAVLT, FAB, STEP weight and STEP total; improvements were also observed within the intervention group between T0 and T6, while, on the other hand, controls showed no advances (Table 3).

Also, the neopterin concentration decreased after supplementation with probiotics, but the reduction in the neopterin concentration cannot be explained by better suppression of HIV replication in CSF, given the fact that HIV-RNA results were undetectable in all participants at T0 and T6. On the other hand, the alteration of the gut mucosa barrier that occurs during HIV-1 infection allows a large amount of bacterial-derived products (such as lipopolysaccharides) to enter into the general circulation, thus causing systemic immune activation. Experimental evidence has shown that lipopolysaccharides can alter the blood-brain barrier permeability [16], possibly leading to leakage of the serum pro-inflammatory milieu into the CSF, precipitating local inflammation. In this sense, several studies have shown that the gut microbiota and its products play central regulatory roles in this bidirectional relationship between the enteric and the central nervous system and on gut-brain axis [17,18,19,20,21,22]. The administration of probiotics to correct dysbiosis reduces microbial translocation, systemic inflammation and, possibly, the subsequent blood-brain barrier permeability, thus exerting an ameliorative effect on intrathecal immune activation and cognitive function.

For these reasons, probiotic supplementation could represent an innovative therapeutic resource, enhancing the direct-indirect effects of the gut environment on the CNS, through the rebalance of the microbiota composition.

## 5. Conclusions

The results of this study were limited by the small sample size of patients analyzed and by the possible influence of the operator and repeated tests on neuropsychological performances. To minimize these practice effects, we used parallel/alternate forms of tests, but we are aware that it does not fully eliminate concerns about this topic. Further limits were the lack of CSF samples at T6 in the control group and of plasmatic/serological markers of immune activation. The significance of our results was restricted by the inclusion of only patients with ANI (Asymptomatic Neurocognitive Impairment). Moreover, a possible limitation is the role of ANI that is, at present, being debated.

Despite these limitations, justified by the complexity of the procedures and ethical issues, our findings suggest that supplementation with probiotics could represent a novel strategy to manage neurocognitive impairment in cART treated HIV-1 infected patients, but larger studies are needed prior to introducing this intervention into everyday clinical practice.

## Figures and Tables

**Table 1:** Sub-study population (supplemented with multi-strain probiotics) and control group main characteristics (expressed as median values; interquartile range is reported between brackets).

Characteristics	Probiotics Supplementation Group	Control Group	*p*-Value
N of subjects	9	26	
Males	9	24	
Females	0	2	
Age	45 (35–52.5)	43 (38.2–53)	0.097
Years from diagnosis	14 (5–19.5)	12.5 (7–23)	0.593
Years on ARV treatment	14 (6.5–16)	12.5 (7–20)	0.373
T CD4 nadir	180 cell/μL (40–438)	288 cell/μL (57–407)	0.678
T CD4 at enrollment	651 cell/μL (563–883)	526 cell/μL (340–663)	0.515
CPE score	7 (7–7.25)	7 (7–8)	0.527
HIV-RNA in CSF	<37 copies/mL	<37 copies/mL	-
Neopterin in CSF	34.14 nmol/L (22.5–65.2)	12.3 nmol/L (10.1–14.8)	0.008

Abbreviations. ARV: antiretroviral; CD4: cluster of differentiation 4; CPE: Central Nervous System Penetration Effectiveness; CSF: cerebrospinal fluid.

**Table 2:** Baseline neurocognitive tests results (expressed as median values; interquartile range is reported between brackets).

Neurocognitive Tests	Probiotics Supplementation Group (T0)	Control Group (T0)	*p*-Value
Rey–Osterrieth Complex Figure immediate recall (*more is better*)	16.6 (15.9–17.8)	13.1 (8.5–22.0)	0.860
Rey–Osterrieth Complex Figure delayed recall (*more is better*)	15.5 (14.3–17.8)	11.6 (6.3–19.9)	0.280
Rey Auditory Verbal Learning Test immediate recall (*more is better*)	46.0 (29.4–47.4)	30.6 (27.9–40.0)	0.214
Rey Auditory Verbal Learning Test delayed recall (*more is better*)	9.2 (5.6–10.9)	5.2 (3.5–8.0)	0.360
Rey Auditory Verbal Learning Test recognition (*more is better*)	98.0 (90.0–100.0)	96.0 (92.0–98.0)	0.400
Verbal Fluency (*more is better*)	15.0 (13.7–16.0)	15.9 (13.9–18.0)	0.314
Phonological Verbal Fluency (*more is better*)	30.0 (23.6–39.2)	26.7 (21.6–35.1)	0.906
Semantic Verbal Fluency (*more is better*)	47.0 (33.5–57.5)	39.0 (33.0–42.0)	0.173
Visual Search Test (*more is better*)	46.2 (45.1–60.0)	46.7 (40.2–50.6)	0.374
Test of Weights and Measures Estimation—Time (*more is better*)	19.0 (13.0–23.5)	22.0 (19.0–24.0)	0.074
Test of Weights and Measures Estimation—Weight (*more is better*)	19.0 (14.0–20.5)	19.0 (16.5–21.0)	0.933
Test of Weights and Measures Estimation—Total (*more is better*)	38.0 (30.5–46.5)	40.0 (36.5–43.5)	0.594
Raven’s Standard Progressive Matrices (*more is better*)	27.5 (22.6–31.6)	28.3 (25.7–31.8)	0.327
Verbal Span forward (*more is better*)	5.0 (3.5–5.7)	5.2 (4.9–6.0)	0.065
Verbal Span backward (*more is better*)	5.0 (4.0–5.0)	4.0 (3.0–4.5)	0.161
Corsi Block Tapping Test forward (*more is better*)	4.7 (4.0–5.2)	5.5 (4.7–6.0)	0.078
Corsi Block Tapping Test backward (*more is better*)	4.0 (3.0–4.0)	5.0 (4.0–6.0)	0.196
Aachener Aphasia Test (*more is better*)	9.0 (9.0–9.0)	9.0 (9.0–9.0)	1.000
Trail Making Test A (s) (*less is better*)	50.0 (44.0–62.0)	50.0 (41.0–67.0)	0.575
Trail Making Test B (s) (*less is better*)	115.0 (93.0–142.0)	97.0 (77.5–144.5)	0.086

**Table 3:** Neurocognitive tests results after supplementation with probiotics (expressed as median values; interquartile range is reported between brackets).

Performed Neurocognitive Tests	Probiotics Supplementation Group (T0 vs. T6)	Control Group (T0 vs. T6)	Probiotics Supplementation Group vs. Control Group (T6 vs.T6)
Rey–Osterrieth Complex Figure immediate recall (*more is better*)	16.6 vs. 22.0 (*p* = 0.007)	13.1 vs. 14.7 (*p* = 0.603)	22.0 (19.0–23.7) vs. 14.7 (8.8–20.3) (*p* = 0.011)
Rey-Osterrieth Complex Figure delayed recall (*more is better*)	15.5 vs. 22.4 (*p* = 0.008)	11.6 vs. 12.6 (*p* = 0.369)	22.4 (22.0–25.5) vs. 12.6 (5.7–19.1) (*p* = 0.011)
Rey Auditory Verbal Learning Test immediate recall (*more is better*)	46.0 vs. 53.0 (*p* = 0.028)	30.6 vs. 32.5 (*p* = 0.619)	53.0 (49.3–55.6) vs. 32.5 (28.7–37.5) (*p* = 0.008)
Rey Auditory Verbal Learning Test delayed recall (*more is better*)	9.2 vs. 12.0 (*p* = 0.034)	5.2 vs. 5.3 (*p* = 0.241)	12 .0 (10.7–13.8) vs. 5.3 (4.3–8.0) (*p* = 0.008)
Rey Auditory Verbal Learning Test recognition (*more is better*)	98.0 vs. 99.0 (*p* = 0.176)	96.0 vs. 96.0 (*p* = 0.575)	99.0 (97.0–100.0) vs. 96.0 (92.0–98.0) (*p* = 0.013)
Verbal Fluency (*more is better*)	15.0 vs. 15.9 (*p* = 0.233)	15.9 vs. 15.3 (*p* = 0.152)	15.9 (14.1–18.0) vs. 15.3 (13.7–16.8) (*p* = 0.594)
Phonological Verbal Fluency (*more is better*)	30.0 vs. 44.0 (*p* = 0.028)	26.7 vs. 25.9 (*p* = 0.271)	44.0 (42.5–45.0) vs. 25.9 (21.6–36.1) (*p* = 0.021)
Semantic Verbal Fluency (*more is better*)	47.0 vs. 49.0 (*p* = 0.373)	39 vs. 38.0 (*p* = 0.396)	49.0 (46.0–49.0) vs. 38.0 (33.5–43.5) (*p* = 0.123)
Visual Search Test (*more is better*)	46.2 vs. 49.0 (*p* = 0.859)	46.7 vs. 46.7 (*p* = 1.000)	49.0 (45.6–50.0) vs. 46.7 (40.2–50.6) (*p* = 0.722)
Test of Weights and Measures Estimation—Time (*more is better*)	19.0 vs. 23.0 (*p* = 0.038)	22.0 vs. 22.0 (*p* = 0.776)	23.0 (21.0–23.5) vs. 22.0 (18.0–25.0) (*p* = 0.512)
Test of Weights and Measures Estimation—Weight (*more is better*)	19.0 vs. 21.0 (*p* = 0.027)	19 vs. 20.0 (*p* = 0.843)	21.0 (20.5–23.5) vs. 20.0 (15.5–21.5) (*p* = 0.08)
Test of Weights and Measures Estimation—Total (*more is better*)	38.0 vs. 45.0 (*p* = 0.138)	40.0 vs. 40.0 (*p* = 0.776)	45.0 (41.5–46.0) vs. 40.0 (35.5–44.0) (*p* = 0.02)
Raven’s Standard Progressive Matrices (*more is better*)	25.7 vs. 30.0 (*p* = 0.208)	28.3 vs. 28.3 (*p* = 0.939)	30.0 (28.5–33.5) vs. 28.3 (25.2–31.6) (*p* = 0.374)
Verbal Span forward (*more is better*)	5.0 vs. 5.0 (*p* = 0.121)	5.2 vs. 5.2 (*p* = 0.632)	5.0 (5.0–6.0) vs. 5.2 (4.6–6.0) (*p* = 0.551)
Verbal Span backward (*more is better*)	5.0 vs. 5.0 (*p* = 1.000)	4.0 vs. 4.0 (*p* = 0.344)	5.0 (4.0–5.0) vs. 4.0 (3.75–5.0) (*p* = 0.206)
Corsi Block Tapping Test forward (*more is better*)	4.7 vs. 5.2 (*p* = 0.049)	5.5 vs. 5.2 (*p* = 0.980)	5.2 (5.0–5.5) vs. 5.2 (5.0–6.0) (*p* = 0.888)
Corsi Block Tapping Test backward (*more is better*)	4.0 vs. 4.0 (*p* = 0.180)	5.0 vs. 5.0 (*p* = 0.317)	4.0 (4.0–4.5) vs. 5.0 (4.0–5.0) (*p* = 0.102)
Aachener Aphasia Test (*more is better*)	9.0 vs. 9.0 (*p* = 1.000)	9.0 vs. 9.0 (*p* = 1.000)	9.0 (9.0–9.0) vs. 9.0 (9.0–9.0) (*p* = 1.000)
Trail Making Test A (s) (*less is better*)	50.0 vs. 43.0 (*p* = 0.041)	50.0 vs. 51.0 (*p* = 0.747)	43.0 (39.0–53.0) vs. 51.0 (40.5–65.5) (*p* = 0.674)
Trail Making Test B (s) (*less is better*)	115.0 vs. 120.0 (*p* = 0.726)	97.0 vs. 98.0 (*p* = 0.279)	120.0 (76.0–138.0) vs. 98.0 (78.5–146.0) (*p* = 0.138)
